# Routine vaccination coverage in low- and middle-income countries: further arguments for accelerating support to child vaccination services

**DOI:** 10.3402/gha.v6i0.20343

**Published:** 2013-04-30

**Authors:** Wenjing Tao, Max Petzold, Birger C. Forsberg

**Affiliations:** 1 Health Systems and Policy Research Group, Department of Public Health Sciences, Karolinska Institutet, Stockholm, Sweden; 2 Centre for Applied Biostatistics, University of Gothenburg, Gothenburg, Sweden; 3 Department of Molecular Medicine and Surgery, Karolinska Institutet, Stockholm, Sweden

**Keywords:** vaccination, immunization programs, immunization schedule, developing countries, child, infant, data collection, health care surveys, World Health Organization

## Abstract

**Background and objective:**

The Expanded Programme on Immunization was introduced by the World Health Organization (WHO) in all countries during the 1970s. Currently, this effective public health intervention is still not accessible to all. This study evaluates the change in routine vaccination coverage over time based on survey data and compares it to estimations by the WHO and United Nations Children's Fund (UNICEF).

**Design:**

Data of vaccination coverage of children less than 5 years of age was extracted from Demographic and Health Surveys (DHS) conducted in 71 low- and middle-income countries during 1986–2009. Overall trends for vaccination coverage of tuberculosis, diphtheria, tetanus, pertussis, polio and measles were analysed and compared to WHO and UNICEF estimates.

**Results:**

From 1986 to 2009, the annual average increase in vaccination coverage of the studied diseases ranged between 1.53 and 1.96% units according to DHS data. Vaccination coverage of diphtheria, tetanus, pertussis, polio and measles was all under 80% in 2009. Non-significant differences in coverage were found between DHS data and WHO and UNICEF estimates.

**Conclusions:**

The coverage of routine vaccinations in low- and middle-income countries may be lower than that previously reported. Hence, it is important to maintain and increase current vaccination levels.

It is estimated that vaccinations prevent 2.5 million child deaths per year (1). If existing vaccines were made available to 90% of the global under-five population, an additional 2 million lives would be saved (1). Immunization plays an important role in achieving the United Nations Millennium Development Goal 4 of reducing under-five mortality by two-thirds between 1990 and 2015.

Through the Expanded Programme on Immunization (EPI) initiated by the World Health Organization (WHO) in 1974, vaccinations against six target diseases (measles, polio, diphtheria, tetanus, pertussis and tuberculosis) were adopted in national immunization programmes across the world. The program resulted in a significant increase in global vaccination coverage that leveled off during the 1990s, as increasing vaccination rates became more difficult with higher vaccination coverage. In fact, many African and South East Asian countries faced a decline in national vaccination coverage during this period, partly due to reduced funding (2). New immunization goals, endorsed by the WHO and United Nations Children's Fund (UNICEF), were set up in 2005 to increase and sustain national vaccination coverage to at least 90% by 2015 (3).

National vaccination coverage is provided regularly by all countries to the WHO and UNICEF. The reported vaccination coverage by each country is commonly based on administrative data from the vaccination service providers. However, it has been shown that the reporting system from the health units to the central level is often not functioning well enough (4). Administrative data has also been criticized for being overestimated compared to household survey data (5). Since 2000, WHO and UNICEF also publish estimations of national and global vaccination coverage that are based on a combination of administrative data and household surveys, and include surveys from the Demographic and Health Surveys (DHS), EPI and UNICEF (6). In spite of adjustment of the nationally reported vaccination coverage with survey data, a study by Lim et al. indicates that there is a remaining discrepancy in vaccination coverage between household surveys and the WHO and UNICEF estimations in low- and middle-income countries (7). Thus, the real vaccination coverage might be lower than what is officially reported.

Previous studies comparing vaccination coverage between administrative and survey data have mainly focused on vaccination against diphtheria, tetanus and pertussis. Little is known about how data sources compare for the other vaccinations, especially in low- and middle-income countries. Thus, the primary objective of this study was to estimate the vaccination coverage over time for all the diseases initially targeted by the EPI in low- and middle-income countries, based on data from DHS. Secondly, we compared the results to the estimations of vaccination coverage made by the WHO and UNICEF, which are commonly referred to as official vaccination coverage figures.

## Methods

DHS are household-based surveys conducted in nationally representative samples. In households, women aged 15–49 years are interviewed on reproductive health, child health and nutrition. Within a country, DHS are ideally conducted every 5 years. Use of standardized core questionnaires in DHS allows for comparisons across countries and time (8).

Information on Bacille Calmette-Guerin (BCG), measles, diphtheria–tetanus–pertussis (DTP) and polio vaccination during the first year of life was retrieved from DHS in low- and middle-income countries between 1986 and 2009, and for which data was available in November 2010 (9). Totally, we found 175 surveys from 71 countries (Table 1). For DTP and polio, the proportion of children who had received three doses was used. In addition, data was also obtained for children who had received all four vaccinations during their first year of life, i.e. fully immunized children, and for children who had received none of the four vaccinations, i.e. non-immunized children.

**Table 1:** Total number of Demographic and Health Surveys (DHS) and number of countries surveyed between 1986 and 2009 in each region as classified by DHS

Region	No. of countries	No. of surveys
Central Asia	4	5
Latin America and Caribbean	11	32
North Africa/West Asia/Europe	8	19
South and Southeast Asia	10	27
Sub-Saharan Africa	38	92

In the surveys, mothers were asked to show the interviewer the vaccination cards of children born up to 4 years before the survey. If no vaccination card could be presented, the mother was asked to recall the vaccinations and the number of doses received. Children who received vaccination during their first year of life were included, irrespective of documentation type, i.e. both vaccination cards and mother's report were accepted. The proportion of households with vaccination cards in the 175 DHS surveys ranged from 4 to 94%, with a median value of 60%. We used the responses for children aged 12–23 months to estimate the vaccination coverage 1 year before the survey. For children aged 24–35 months, we estimated the vaccination coverage 2 years before the survey. For children aged 36–47 months, we estimated the vaccination coverage 3 years before the survey, and for children aged 48–59 months, we estimated the vaccination coverage 4 years before the survey.

Average trends for low- and middle-income countries, presented as % units for each of the outcomes, were estimated using random coefficient regression with identity link. Using graphical residual analysis, it was found that a linear model with a country-specific random intercept, but a common slope, fitted the data appropriately in terms of homoscedasticity and normal distribution. Each country was considered an independent unit of observation. WHO and UNICEF estimates of vaccination coverage were obtained from the corresponding countries and time period for comparison (10). Differences in trends between DHS-reported vaccination coverage and WHO and UNICEF–estimated vaccination coverage were tested by estimating an interaction term between year and data source. The model also allowed for differences in intercept between the two trends.

In a separate analysis, we estimated what the discrepancy in vaccination coverage between DHS and the WHO/UNICEF would correspond to in absolute numbers of children during a limited time-period from 2001 to 2009. The number of children aged 12–23 months in low- and middle-income countries for a given year was multiplied with the differential between the DHS and WHO/UNICEF trend line for the previous year. The population was estimated from birth rate, infant mortality rate and under-five mortality rate obtained from the International Data Base of the United States Census Bureau in low- and middle-income countries (11).

## Results

### Proportion of children vaccinated with BCG

**Fig. 1:**
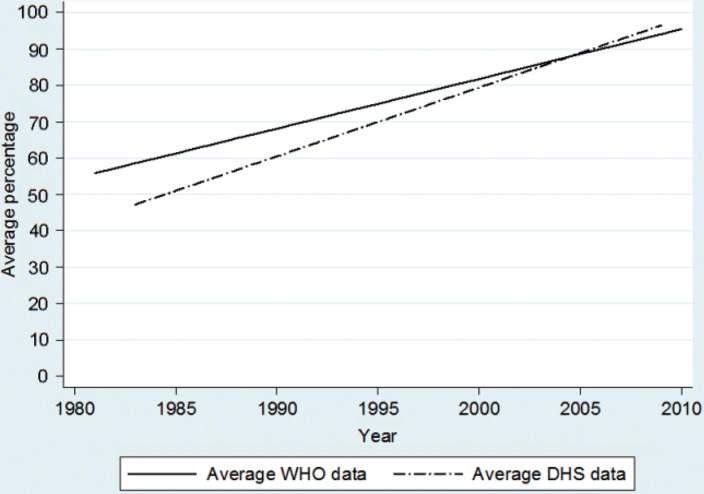
Estimated mean trend in BCG coverage of children aged 12–23 months. Data is obtained from 175 DHS surveys from 71 low- and middle-income countries and compared with WHO-estimated coverage for the corresponding countries from 1986 to 2009.

The overall trend over time for tuberculosis vaccination (BCG) according to DHS data was estimated as an annual increase of 1.96% units (*p*<0.001) in the surveyed countries from 1986 to 2009 (Fig. 1). In 2009, the DHS trend line suggests 97% coverage and WHO and UNICEF suggests 94% coverage. The difference between these two trends was statistically significant (*p*=0.001).

### Proportion of children vaccinated with DTP3

**Fig. 2:**
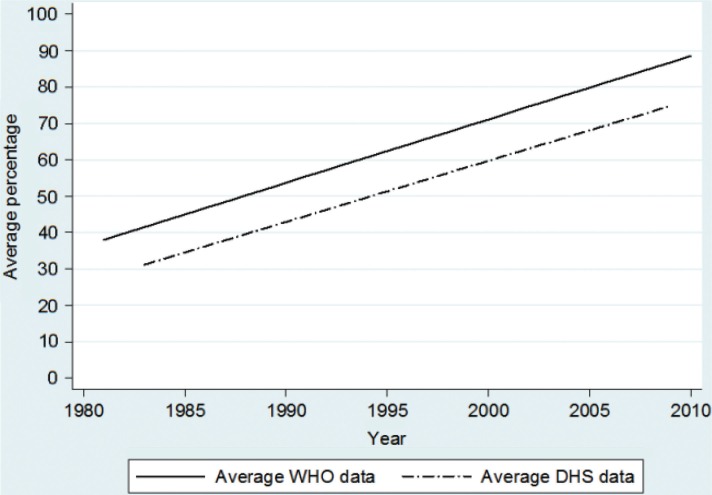
Estimated mean trend in DTP3 coverage of children aged 12–23 months. Data is obtained from 175 DHS surveys from 71 low- and middle-income countries and compared with WHO-estimated coverage for the corresponding countries from 1986 to 2009.

The overall trend over time for the third dose of diphtheria, tetanus and pertussis vaccination (DTP3) was estimated as an annual increase of 1.80% units (*p*<0.001) in the surveyed countries from 1986 to 2009 (Fig. 2). In 2009, the DHS trend line suggests 77% coverage and WHO and UNICEF suggests 87% coverage. The difference in these trends was not statistically significant (*p*=0.608).

### Proportion of children vaccinated with three doses of polio

**Fig. 3:**
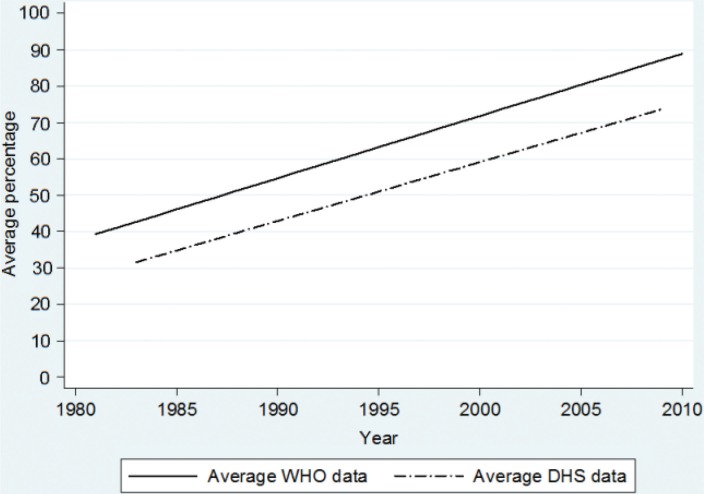
Estimated mean trend in polio coverage of children aged 12–23 months. Data is obtained from 175 DHS surveys from 71 low- and middle-income countries and compared with WHO-estimated coverage for the corresponding countries from 1986 to 2009.

The overall trend over time for the third dose of polio was estimated as an annual increase of 1.75% units (*p*<0.001) in the surveyed countries from 1986 to 2009 (Fig. 3). In 2009, the DHS trend line suggests 76% coverage and WHO and UNICEF suggests 87% coverage. The difference in these trends was not statistically significant (*p*=0.835).

### Proportion of children vaccinated with measles

**Fig. 4:**
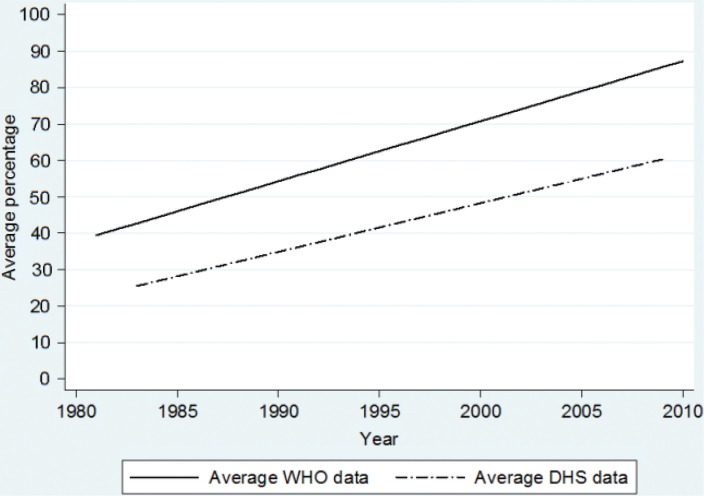
Estimated mean trend in measles coverage of children aged 12–23 months. Data is obtained from 175 DHS surveys from 71 low- and middle-income countries and compared with WHO-estimated coverage for the corresponding countries from 1986 to 2009.

The overall trend over time for measles vaccination was estimated as an annual increase of 1.53% units (*p*<0.001) in the surveyed countries from 1986 to 2009 (Fig. 4). In 2009, the DHS trend line suggests 50% coverage and WHO and UNICEF suggests 73% coverage. The difference in these trends was not statistically significant (*p*=0.112).

### Proportion of children fully immunized

Children aged 12–23 months who have received one dose of BCG and measles and three doses of DTP and polio are regarded as fully immunized. The overall trend over time was estimated from the DHS surveys as an annual increase of 1.08% units (*p*<0.001) in the surveyed countries from 1986 to 2009. In 2009, the DHS trend line suggests 46% coverage. No data from WHO and UNICEF was available for comparisons.

### Proportion of non-immunized children

In this study, children aged 12–23 months who have not received any of the vaccinations belonging to the original EPI, i.e. BCG, measles, three doses of DTP or three doses of polio, are classified as non-immunized. The overall trend over time in this variable was estimated as an annual decrease of 1.95% units (*p*<0.001) in the surveyed countries from 1986 to 2009. The DHS data suggests that by 2007, all children aged 12–23 months in low- and middle-income countries have already received at least one of the EPI vaccinations during their first year of life. No data from WHO and UNICEF was available for comparisons.

### Data for the total population surveyed

Between 1986 and 2009, a total of 801,382 children under 5 years of age were included in the DHS surveys. Out of them, 78% received BCG and 59% received DTP3 and polio, whereas only 50% received measles vaccination. Furthermore, 35% of the children included in the surveys obtained all four vaccinations, but 19% did not receive any of the included vaccinations in this study. If WHO data were applied to the same population, the results would have been 85% for BCG, 74% for polio and 73% for DTP3 and measles (Table 2).

**Table 2:** Vaccination coverage (%) for BCG, DTP, polio, measles, none and full routine immunization of children during their first year of life, as estimated in DHS surveys from 1986 to 2009

			Vaccination coverage (percent)								
						
	Population	BCG		DTP3		Pol3		Measles		Fully vaccinated^b^	None^c^
Year	12–23 months^a^	DHS	WHO	DHS	WHO	DHS	WHO	DHS	WHO	DHS	DHS
1986	17 425	52	62	36	46	36	47	30	47	21	42
1987	19 143	54	64	38	48	38	49	32	49	22	40
1988	23 250	56	65	39	50	39	51	33	50	23	38
1989	27 145	58	66	41	52	41	53	35	52	24	36
1990	37 702	60	68	43	54	43	54	36	54	25	34
1991	32 922	62	69	45	55	45	56	38	55	26	32
1992	32 557	64	71	47	57	46	58	40	57	27	30
1993	26 776	66	72	48	59	48	60	41	59	28	28
1994	29 178	68	73	50	61	50	61	43	61	30	26
1995	35 830	70	75	52	62	52	63	44	62	31	24
1996	33 823	72	76	54	64	53	65	46	64	32	22
1997	54 326	74	77	56	66	55	67	47	66	33	20
1998	53 497	76	79	57	68	57	68	49	67	34	18
1999	38 810	78	80	59	69	59	70	50	69	35	16
2000	46 078	80	82	61	71	60	72	52	71	36	14
2001	37 403	82	83	63	73	62	73	53	72	37	12
2002	55 472	84	84	65	75	64	75	55	74	38	10
2003	59 558	85	86	66	76	66	77	56	76	39	8
2004	60 553	87	87	68	78	67	79	58	77	40	6
2005	62 817	89	89	70	80	69	80	59	79	41	5
2006	37 521	91	90	72	82	71	82	61	81	43	3
2007	26 007	93	91	74	83	73	84	62	82	44	1
2008	13 408	95	93	75	85	74	86	64	84	45	0
2009	<1	97	94	77	87	76	87	66	86	46	0
**1986–2009**	**801** **382**	**78**	**85**	**59**	**73**	**59**	**74**	**50**	**73**	**35**	**19**

a Number of children included in the surveys.

b Vaccinated with BCG, measles and three doses of DTP and polio.

c Received no vaccination with BCG, measles or third dose of DTP and polio.

If our results were applicable to all low- and middle-income countries, the discrepancy in vaccination coverage between DHS and WHO/UNICEF data would correspond to a reduction in the number of vaccinated children aged 12–23 months with 100 million for DTP, 114 million for polio and 197 million for measles during 2000–2009.

## Discussion

Based on the data from DHS, the vaccination coverage of DTP, polio and measles was below 80% in low- and middle-income countries in 2009, whereas BCG achieved 97% coverage during the same year. The difference in vaccination schedule for routine vaccinations in the EPI may contribute to the variation in coverage. BCG is usually given at birth and can be provided when the mother is in contact with health care for delivery, whereas measles vaccination is not given until 9 months of age or later. The trend lines for DTP and polio follow each other closely. DTP and polio vaccinations are given in a three-dose regime and usually at the same time when the child is in contact with health services. This could explain similarities in the two trend lines.

The coverage of measles vaccination is poor. Half of the children in the DHS surveys were vaccinated against measles in 2009. The data for measles vaccinations is based on the first dose given. Nowadays, it is recommended that all children be given a second opportunity for measles immunization, either through a routine two-dose schedule or through supplementary immunization activities (12). Countries now gradually adopt the two-dose strategy and this may accelerate measles protection more than what is reflected in our results. Data from DHS suggests that less than half of the children in low- and middle-income countries in 2009 were fully vaccinated according to the original EPI, i.e. measles, polio, diphtheria, tetanus, pertussis and tuberculosis. In contrast, all children have received at least one of the vaccinations, indicating that immunization services are available but incompletely utilized and delivered. Multiple factors may complicate immunization efforts in low- and middle-income settings and contribute to underutilization of resources. A recent systematic review identifies problems related to the immunization systems, communication and information, family characteristics and parental attitudes and knowledge (13). In cross-national studies, inferior immunization outcomes have been associated with several development indicators, such as low female literacy and poor governance (14). Although vaccinations are given, there may be substantial delays in providing them compared to the recommended age of vaccination. This may reduce the effectiveness of the vaccine and increase the burden of disease (15, 16). The introduction of new vaccinations does not seem to influence the performance of routine immunization programs (17), suggesting that performances are more dependent on a well-functioning immunization service than the number of vaccines provided.

Previous studies have indicated that low- and middle-income countries have a tendency to inflate the reported national vaccination coverage, represented by the third dose of DTP, to WHO and UNICEF as compared to household survey data (5, 7). Our study compared survey data from DHS to the WHO and UNICEF estimates of vaccination coverage for the corresponding country and year. The results suggest that the WHO and UNICEF estimates of vaccination coverage for DTP, polio and measles were higher than survey data, but the difference was not statistically significant. Our data was insufficient for country-level comparisons. In order to pinpoint the countries and regions where differences between sources are particularly large, future studies adopting a country and regional approach would be of interest.

The WHO and UNICEF estimates are, along with the reported vaccination coverage by country, commonly regarded as the official vaccination coverage. The estimates are based on reviews of available data by country, usually a combination of administrative data from routine vaccination systems and household surveys from the DHS, EPI or UNICEF (6). Thus, DHS data and WHO and UNICEF estimates are not completely independent, which may contribute to the lack of statistical significance. In contrast to previous studies, WHO and UNICEF estimates of vaccination coverage do not appear to be statistically significantly inflated compared to survey data. However, our results indicate a worrisome difference of 10–20% in the reported vaccination coverage between these two sources. If these findings are accurate, it would reduce the number of vaccinated children in low- and middle-income countries with 100–200 million during 2000–2009 compared to the WHO and UNICEF estimates. Furthermore, it would also indicate that we are lagging behind the targets set by the WHO and UNICEF to achieve at least 90% vaccination coverage by 2015, and the goal of eradicating measles in five out of six WHO regions (18). Thus, accurate monitoring of vaccination coverage is of high public health importance.

The methodology used in this study has some limitations. Our results depend on the quality of DHS data. DHS is the largest program for the collection of quantitative data on population and health from households in low- and middle-income countries and is generally considered as one of the most reliable sources of maternal and child health data (8). The DHS program has spent vast resources on developing survey instruments, guidelines, careful selection of surveyors, training in survey techniques and analysis of collected data (19). Unlike most administrative data, survey data incorporates vaccinations provided by the private sector. However, the length and complexity of the questionnaires used in the surveys may adversely affect the accuracy of the responses.

Between 1986 and 2009, a total of 175 DHS surveys were conducted in 71 low- and middle-income countries. The countries were not randomly selected and the surveys were not repeated with the same frequency in all countries. Thus, some countries are overrepresented in the aggregated DHS data, resulting in selection bias. Furthermore, low-income countries especially in Sub-Saharan Africa are overrepresented in the DHS surveys, leading to a probable underestimation of the true vaccination coverage in low- and middle-income countries.

Previous studies have also raised the issue of recall and reporting bias in surveys (20–22). The immunization data in DHS surveys is collected from two sources: vaccination cards and mothers’ report. Using data from both mothers’ reports and vaccination cards is more inclusive than vaccination cards alone. Several studies have evaluated the quality of information from mothers’ report. Some of them suggest high validity of mother's recall, whereas other studies are pointing to significant recall errors resulting in either over- or underestimation (23–26). A recent systematic review of 45 studies assessed the validity of vaccination cards and parental recall to estimate vaccination coverage through comparison with a medical provider source. Vaccination cards tended to underestimate vaccination coverage, whereas parental recall more commonly overestimated coverage. Combining cards and recall resulted in overestimation of coverage in some studies and understimation in others. Overall, the study found poor agreement between vaccination information from households and medical providers (27). The results from the systematic review are primarily based on studies from high-income countries, and may not be directly transferable to low- and middle-income settings.

In spite of several potential sources of errors, DHS is considered to be one of the best sources of population-based information on health and health service utilization in low- and middle-income countries (5). Thus, we consider vaccination coverage based on DHS data to be of adequate quality and validity to which other measurement of the same variables can be compared.

In conclusion, our study indicates that the coverage of routine vaccinations in low- and middle-income countries may be inadequate to meet the targets set by the WHO and UNICEF to achieve at least 90% vaccination coverage by 2015. Our results provide strong support for further efforts to improve current vaccination levels and to optimize the use of existing resources. High quality data to measure progress is needed and should be prioritized at national and global levels. Improved program monitoring is vital in identifying populations at risk of vaccine coverage failures and epidemics.
